# Association of Apolipoprotein E Polymorphism with Adipokines and Cardiovascular Disease Risk in Rheumatoid Arthritis Patients

**DOI:** 10.3390/life10120330

**Published:** 2020-12-07

**Authors:** Yi-Ming Chen, Po-Ku Chen, Ching-Kun Chang, Chi-Chen Lin, Hsin-Hua Chen, Joung-Liang Lan, Shih-Hsin Chang, Der-Yuan Chen

**Affiliations:** 1 Division of Allergy, Immunology and Rheumatology, Department of Medical Research, Taichung Veterans General Hospital, Taichung 40705, Taiwan; ymchen1@vghtc.gov.tw (Y.-M.C.); shc5555@hotmail.com (H.-H.C.); 2 Ph.D. Program in Translational Medicine & Rong Hsing Research Center for Translational Medicine, National Chung Hsing University, Taichung 40227, Taiwan; lincc@dragon.nchu.edu.tw; 3 Faculty of Medicine, National Yang-Ming University, Taipei 11221, Taiwan; 4 Rheumatology and Immunology Center, China Medical University Hospital, Taichung 40447, Taiwan; pago99999@gmail.com (P.-K.C.); kun80445@gmail.com (C.-K.C.); jounglancmuh@gmail.com (J.-L.L.); sherry61976@hotmail.com (S.-H.C.); 5 Translational Medicine Laboratory, China Medical University Hospital, Taichung 40447, Taiwan; 6 College of Medicine, China Medical University, Taichung 40447, Taiwan; 7 Rheumatic Diseases Research Center, China Medical University Hospital, Taichung 40447, Taiwan; 8 Research and Development Center for Immunology, China Medical University, Taichung 40447, Taiwan

**Keywords:** apoE genotypes, lipid profile, adipokines, cardiovascular disease (CVD), rheumatoid arthritis (RA)

## Abstract

Apolipoprotein E (ApoE) polymorphism and adipokines are linked to atherosclerosis. We aimed to investigate the associations of apoE genotypes with adipokines, inflammatory parameters, and cardiovascular disease (CVD) risks in rheumatoid arthritis (RA) patients. We enrolled 152 RA patients and 49 healthy control (HC) subjects. The apoE genotyping was determined by a polymerase chain reaction, while plasma levels of adipokines and inflammatory cytokines were measured with ELISA. Although apoE genotypes distributions were indistinguishable between RA patients and HC, we found significantly higher levels of apoE and adipokines in RA patients compared with HC. RA patients with ε2ε3 genotype had lower levels of TNF-α, IL-6, resistin, and visfatin, but higher leptin levels compared with ε3ε3 genotype patients. Patients with ε3ε4 genotype had significantly higher low-density lipoprotein-cholesterol (LDL-C) levels and atherogenic index scores compared with ε2ε3 genotype carriers. Moreover, patients with ε2ε3 genotype had significantly lower 10-year CVD risk than ε3ε3 or ε3ε4 genotype patients. ε3ε4 genotype and adiponectin levels were independent predictors of a high 10-year CVD risk. RA patients with ε2ε3 genotype are associated with lower levels of TNF-α, IL-6, resistin, visfatin, and CVD risk, while RA patients with ε3ε4 genotype exhibited higher levels of LDL-C, insulin resistance, and higher CVD risks.

## 1. Introduction

Atherosclerosis, a chronic inflammatory vascular disease characterized by atheromatous plaque buildup, is associated with an elevated risk of cardiovascular events [1]. Rheumatoid arthritis (RA) is an inflammatory disease that can lead to chronic synovitis, cartilage damage, and joint destruction [2,3]. Therefore, RA is commonly associated with accelerated atherosclerosis and increased risk of cardiovascular disease (CVD) [4,5,6]. Although the pathogenesis of accelerated atherosclerosis in RA is not fully understood, the high CVD burden could be explained by traditional CVD risk factors and chronic inflammation in this disease [7,8].

The involvement of proinflammatory cytokines such as tumor necrosis factor-α (TNF-α) and interleukin (IL)-6 in RA pathogenesis [9,10] has been supported by the effectiveness of biologics targeting these cytokines [11]. Recent studies also identified TNF-α and IL-6 as the pathogenic cytokines in atherosclerosis in rheumatic patients and the general population [12,13]. Through promoting deterioration of lipid profile and insulin resistance (IR), both cytokines are traditional risk factors of atherosclerosis [12,13]. White adipose tissue alters adipokine secretion profile and then participates in inflammatory responses [14,15], which plays a significant role in atherogenesis and IR. Adiponectin, leptin, resistin, and visfatin are the most widely-explored adipokines responsible for regulating atherosclerosis and inflammatory responses [16]. Although previous studies revealed significantly higher adipokines in RA patients than in healthy subjects [17], the association of adipokine levels with inflammation, IR, or atherosclerosis in RA has not been clearly defined [18].

Apolipoprotein E (ApoE), a component of major lipoprotein classes, not only plays a vital role in the development of atherosclerosis in humans [19,20] but participates in the modulation of immune response and inflammation as well [20,21]. The polymorphic human ApoE gene, located on chromosome 19q13.2 [22], has six different genotypes (ε2/ε2, ε2/ε3, ε2/ε4, ε3/ε3, ε3/ε4, and ε4/ε4) with three common alleles (ε2, ε3, and ε4) encoding the major apoE isoforms, apoE2, apoE3, and apoE4, respectively. ApoE3 polypeptide is the most common isoform. ApoE4 isoform is associated with higher, but apoE2 isoform with lower total cholesterol and low-density lipoprotein-cholesterol (LDL-C) levels [23]. Increasing evidence indicates that the genetic variation of apoE is a determinant of CVD susceptibility, and the apoE4 allele is associated with increased CVD risks [24,25].

In this pilot study, we aimed to (1) evaluate the significance of apoE genotypes as the risk factors of CVD in RA patients; (2) examine the differences in the plasma lipid profile, plasma adipokines levels, IR, and inflammatory parameters in RA patients with different apoE genotypes; and (3) evaluate the association of plasma adipokines levels with lipid profile, IR, RA inflammatory parameters, and CVD risk in RA patients with different apoE genotypes. 

## 2. Material and Methods

### 2.1. Subjects

In this prospective study, 152 RA patients who fulfilled the 2010 classification criteria of the American College of Rheumatology/European League Against Rheumatism collaborative initiative [26] were consecutively enrolled. Patients with a recent history (i.e., within one year before enrollment) of coronary heart disease or ischemic stroke were excluded. Disease activity was assessed using the 28-joint disease activity score (DAS28) [27]. Each RA patient received corticosteroids, nonsteroidal anti-inflammatory drugs, and at least one conventional synthetic disease-modifying anti-rheumatic drug (csDMARD) at an active status. Follow-up for the emergence of CVD, which included acute myocardial infarction and ischemic stroke, was done for at least five years. Forty-nine age- and gender-matched healthy volunteers served as healthy controls (HC). The Institutional Review Board of Taichung Veterans General Hospital approved this study (CF12130), and written consent was obtained from each participant according to the Declaration of Helsinki.

### 2.2. Determination of Plasma Lipid Profiles and Atherogenic Index (AI)

All blood samples were collected from participants in the early morning after an overnight fast for 12 h. Plasma levels of total cholesterol, triglyceride, high-density lipoprotein cholesterol (HDL-C), and LDL-C were measured using enzymatic methods with a chemistry analyzer (Hitachi 7600, Hitachi, Tokyo, Japan) following the manufacturer’s instructions. The AI, the ratio of total cholesterol/HDL-C, was calculated.

### 2.3. Measurements of Insulin Resistance

Serum insulin levels were determined using a commercially available assay kit (IMMULITE, I-2000, EURO/Diagnostic Products Corporation, Gwynedd, UK). Homeostasis model assessment for insulin resistance (HOMA-IR) was calculated using the formula: fasting plasma insulin (μIU/L) × fasting plasma glucose (mmol/L) /22.5 [28].

### 2.4. Measurement of 10-Year Risk of CVD Including QRISK-2 Score 

The global 10-year risk for a heart attack or stroke was estimated by calculating the QRISK-2 scores [29,30]. Briefly, factors including age, sex, ethnicity, physical characteristics, total cholesterol/HDL-C ratio, self-reported smoking status, diabetic status, the presence of kidney disease, and family history of heart disease were considered in determining QRISK-2 score of each RA patient.

### 2.5. Determination of ApoE Genotypes

Genomic DNA samples were extracted from the enrolled subjects’ peripheral blood using Genomic DNA Extraction kits (RBC bioscience, New Taipei City, Taiwan). The apoE genotypes were determined by polymerase chain reaction (PCR)-restriction fragment length polymorphism according to the methods of the previous reports [25,31] with some modification. A 244-base pair (bp) fragment located on exon 4 of the ApoE gene was amplified in a DNA thermal cycler (Pharmacia, Uppsala, Sweden) using the oligonucleotide primers 5′-AGAATTCGCCCCGGCCTGGTACAC-3′(sense) and 5′-TAAGCTTGGCACGGCTGTCCA AGGA-3′ (antisense). These primers were designed to encompass the polymorphic region of amino acids 112 and 158 of the apoE gene. The PCR reaction was carried out for 40 cycles under the following conditions: denaturing at 95 °C for 45 s, annealing at 59 °C for 45 s, and extension at 72 °C for 2 min, with the final cycle extension running for 10 min. The amplified 244-bp product was digested with *Hha*I (Pharmacia, Uppsala, Sweden) overnight at 37 °C and underwent electrophoresis on a 4% Metaphor gel (FMC Products, Rockland, Me) in Tris-acetate-EDTA (TAE) buffer containing 0.5 μg/mL ethidium bromide. The gel patterns obtained for the heterozygous ε2/ε3, ε2/ε4, ε3/ε4 genotypes were a combination of the homozygous fragments. 

### 2.6. Detection of Plasma Levels of ApoE, Adipokines, and Proinflammatory Cytokines

Plasma levels of apoE were detected by using ELISA (Eagle eBioscience, San Diego, CA, USA) (Thermo Fisher Scientific, Waltham, MA, USA). Plasma levels of adiponectin, leptin, and resistin were measured using the Lincoplex Multiplex Immunoassay (EMD Millipore, Waltham, MA, USA), and visfatin by using a visfatin C-terminal enzyme-linked immunosorbent assay kit (Phoenix Pharmaceuticals, Burlingame, CA, USA). Plasma levels of TNF-α, IL-6, and IL-17A were determined using ELISA (PeproTech Inc., Rocky Hill, NJ, USA) according to the manufacturer’s instructions.

### 2.7. Statistical Analysis

Results are presented as the mean ± standard deviation (SD) or median (interquartile range). The Mann–Whitney U test was used for between-group comparison of lipid profiles, adipokines levels, and proinflammatory cytokine levels. We constructed a logistic regression model to evaluate the effects of traditional CVD risk factors, levels of lipid profile, adipokines, and RA inflammatory parameters to predict CVD risk in RA patients. The correlation coefficient was obtained using Spearman’s rank test. A *p*-value < 0.05 was considered significant. 

## 3. Results

### 3.1. Demographic Data and Laboratory Findings in RA Patients and Healthy Control Subjects 

**Figure 1:**
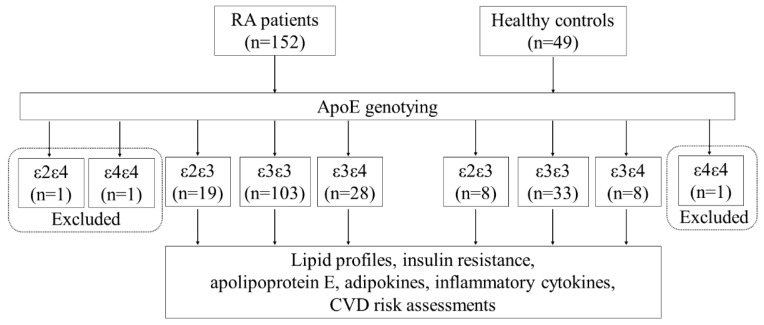
Study design workflow. RA: rheumatoid arthritis; ApoE: apolipoprotein E; CVD: cardiovascular disease.

**Figure 2:**
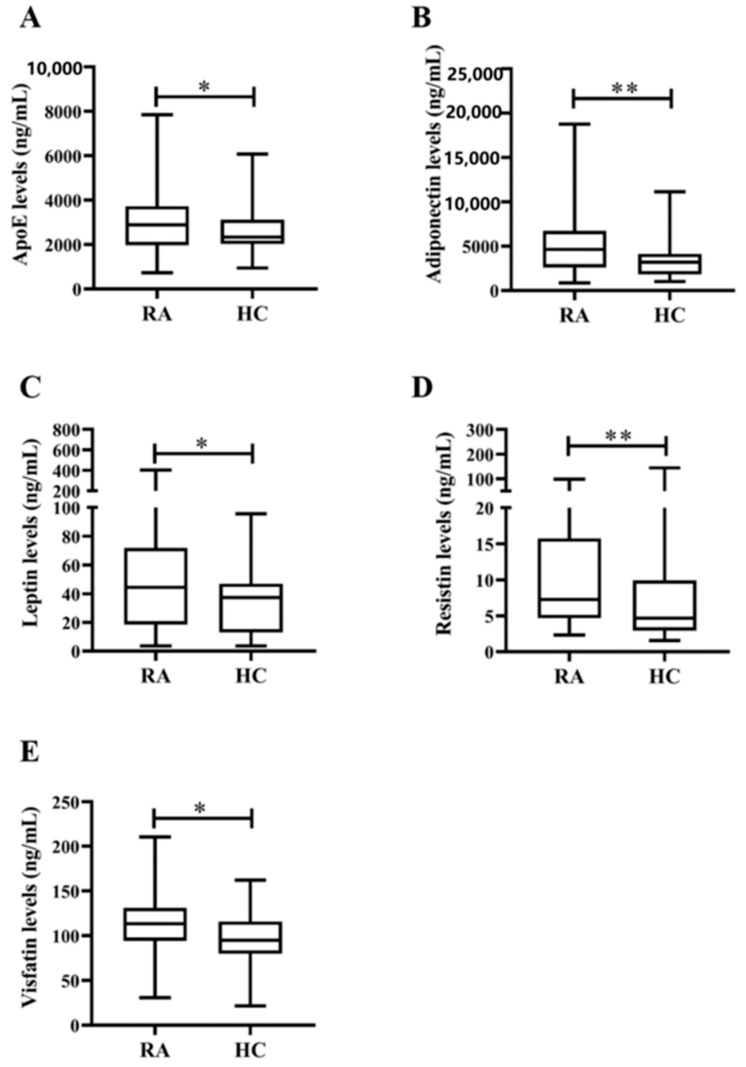
Comparisons of plasma apoE levels and adipokines levels between RA patients and healthy control subjects. Comparisons of plasma levels of (**A**) apoE, adipokines including (**B**) adiponectin, (**C**) leptin, (**D**) resistin, and (**E**) visfatin between rheumatoid arthritis (RA) patients and healthy control (HC) subjects. Data are presented as box-plot diagrams, with the box encompassing the 25th percentile (lower bar) to the 75th percentile (upper bar). The horizontal line within the box indicates the median value, respectively, for each group. * *p* < 0.05, ** *p* < 0.005, versus HC, determined by Mann–Whitney U test.

Figure 1 shows the study design workflow. With the limited case numbers, ε2/ε4 (n = 1) and ε4/ε4 (n = 1) carriers in RA patients and ε4/ε4 (n = 1) in the healthy group were excluded from the analysis. As illustrated in Table 1 and Figure 2A, significantly higher levels of plasma apoE were noted in RA patients compared with HC. Among the examined adipokines, significantly higher adiponectin levels, leptin, resistin, and visfatin were observed in RA patients compared with HC (Figure 2B–E). Plasma IL-17A levels were also significantly higher in RA patients than in HC. However, there was no significant difference in plasma levels of TNF-α or IL-6 between RA patients and HC. Furthermore, there were no significant differences in demographic data between RA patients and HC.

### 3.2. Frequencies and Distribution of ApoE Genotypes in RA Patients and Healthy Controls

**Figure 3:**
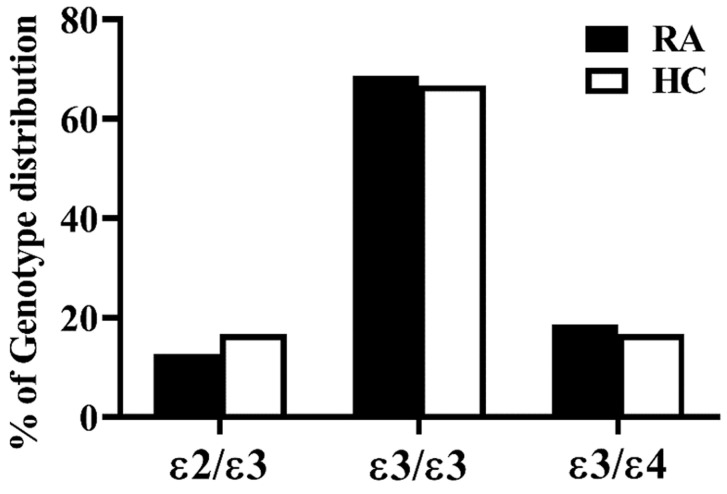
ApoE genotypes distribution between rheumatoid arthritis (RA) patients and healthy control (HC) subjects.

As shown in Figure 3, the distribution of apoE genotypes between RA patients and HC was indistinguishable, with ε3/ε3 the most common apoE genotype. (68.6% in RA patients and 66.6% in HC). 

### 3.3. Clinical Characteristics and Laboratory Findings in RA Patients with Different ApoE Genotypes

**Figure 4:**
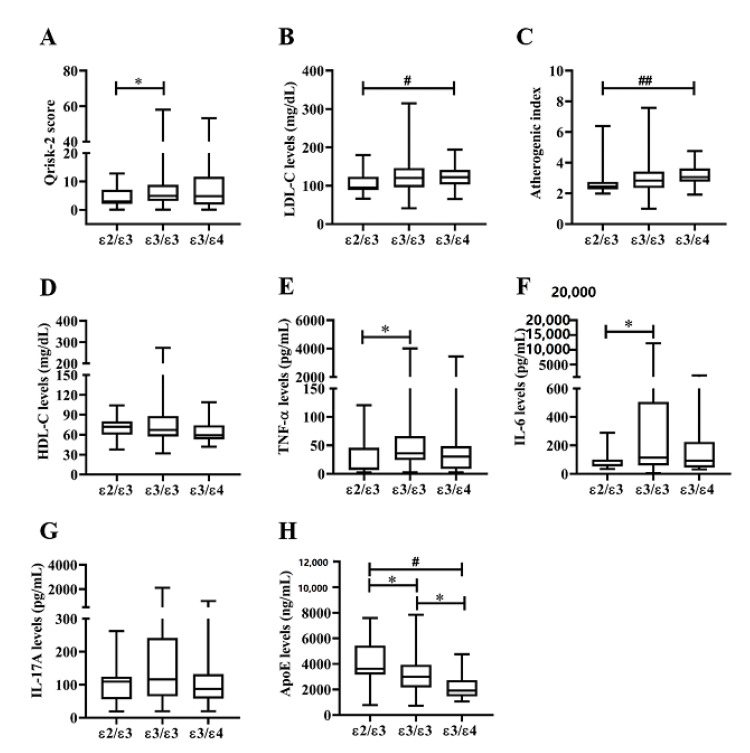
Comparisons of CVD risk, lipid profile, and cytokines in RA patients with different apoE genotypes. Comparisons of (**A**) CVD risk (Qrisk-2 score), (**B**) LDL-C levels, (**C**) atherogenic index, (**D**) HDL-C levels, (**E**) TNF-α levels, (**F**) IL-6 levels, (**G**) IL-17A levels, and (**H**) plasma apoE levels among RA patients with different apoE genotypes. Data are presented as box-plot diagrams, with the box encompassing the 25th percentile (lower bar) to the 75th percentile (upper bar). The horizontal line within the box indicates the median value for each group. * *p* < 0.05, versus patients with apoE ε3ε3 genotype; ^#^ *p* < 0.05, ^##^ *p* < 0.001, versus patients with apoE ε3ε4 genotype, determined by Mann–Whitney U test.

**Figure 5:**
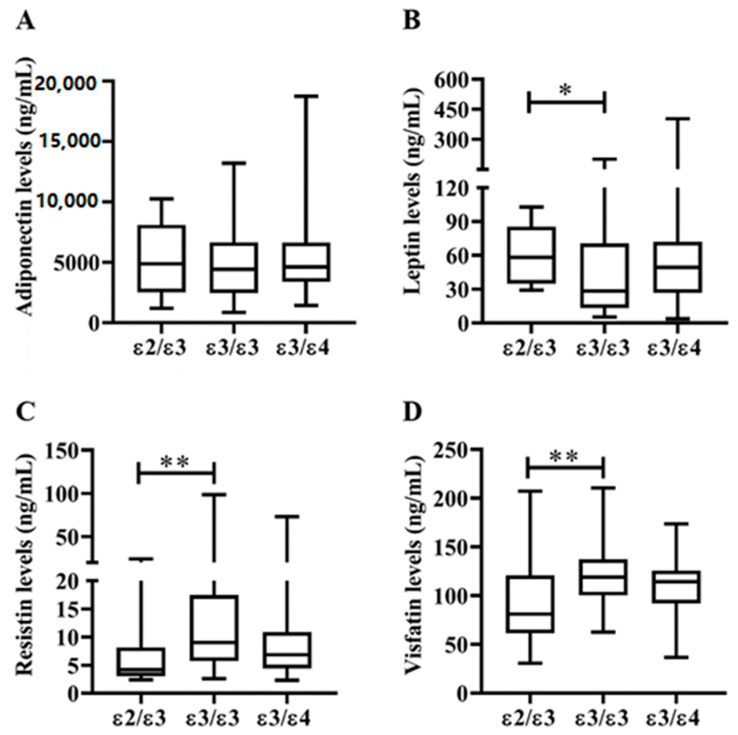
Comparisons of plasma adipokines levels in RA patients with different apoE genotypes. Comparisons of (**A**) adiponectin, (**B**) leptin, (**C**) resistin, and (**D**) visfatin among RA patients with different apoE genotypes. Data are presented as box-plot diagrams, with the box encompassing the 25th percentile (lower bar) to the 75th percentile (upper bar). The horizontal line within the box indicates the median value for each group. * *p* < 0.05, ** *p* < 0.01, versus patients with apoE ε3ε3 genotype, determined by Mann–Whitney U test.

As illustrated in Table 2, there were no significant differences in demographic data, traditional CVD risk factors, BMI, or proportion of seropositivity for rheumatoid factor (RF) or anticitrullinated peptide antibody (ACPA) among RA patients with different apoE genotypes. As shown in Figure 4A, patients with ε2ε3 genotype had lower Qrisk-2 scores for 10-year risk of CAD compared to those with ε3ε3 genotype or ε3ε4 genotype (*p* = 0.011 or *p* = 0.053, respectively). Similarly, a trend of a lower proportion of CVD events was observed in patients with ε2ε3 genotype compared to those with ε3ε4 genotype (5.3% versus 17.9%). Significantly lower levels of LDL-C and atherogenic index were also found in RA patients with ε2ε3 genotype than in those with ε3ε4 genotype (Figure 4B,C). However, there was no significant difference in plasma HDL-C levels among patients with different apoE genotypes. As shown in Table 2 and Figure 4E–F, patients with ε2ε3 genotype had significantly lower levels of TNF-α, IL-6, and C-reactive protein (CRP) compared with ε3ε3 genotype. Although it did not reach statistical significance, patients with the ε2ε3 genotype had lower DAS28 than those with the ε3ε3 genotype. In addition, patients with ε2ε3 genotype had significantly higher levels of plasma apoE compared with ε3ε3 genotype or ε3ε4 genotype carriers (Figure 4H). Among the examined adipokines (Figure 5), significantly lower levels of resistin (Figure 5C) or visfatin (Figure 5D) and higher levels of leptin (Figure 5B) were found in patients with ε2ε3 genotype than in those with ε3ε3 genotype. 

### 3.4. Correlations between Plasma Adipokines Levels and Inflammatory Parameters or Lipid Profiles in RA Patients with Different ApoE Genotypes

As illustrated in Table 3, plasma adiponectin levels were positively correlated with levels IL-17A and HDL-C while being negatively correlated with IR in those with ε3ε3 genotype. Plasma leptin levels were positively correlated with TNF-α levels in patients with ε2ε3 or ε3ε4 genotype and were positively correlated with levels of IL-17A and triglyceride (TG) in those with the ε2ε3 genotype. Plasma resistin levels were positively correlated with IL-6 levels in RA patients with ε2ε3 genotype, and with IR in those with ε3ε4 genotype. Plasma visfatin levels were positively correlated with atherogenic index in patients with the ε2ε3 genotype and with IR and BMI in those with the ε3ε3 genotype. 

### 3.5. Logistic Regression Analysis 

As illustrated in Table 4, univariate regression analysis revealed that ACPA positivity, plasma adiponectin levels, and AI were identified as potential predictors of high Qrisk-2 score, an estimated calculator of global 10-year CVD risk in RA patients. The multivariate regression analysis demonstrated that ε3ε4 genotype and ACPA positivity were positive predictors of high 10-year CVD risk, while plasma adiponectin levels were a negative predictor for CVD risk.

## 4. Discussion

Increased CVD risk in RA patients results from a complex interaction among traditional CV risk factors, systemic inflammation, and genetic components [7,8,32,33]. With apoE genetic variants shown to be related to CVD risk [32,33], we are the first to examine the associations of apoE genotypes with lipid profile, plasma adipokines levels, and CVD risk in the RA population. Although the distribution of apoE genotypes between patients and HC was indistinguishable, we revealed significantly higher levels of plasma apoE and adipokines in RA patients compared with HC. Among RA patients with different apoE genotypes, those with ε2ε3 genotype had significantly lower LDL-C levels and AI scores compared to those with ε3ε4 genotype. Patients with ε2ε3 genotype also had significantly higher plasma levels of apoE and leptin, whereas lower levels of TNF-α, IL-6, resistin, and visfatin compared with ε3ε3 genotype patients. Moreover, multivariate regression analysis revealed that the ε3ε4 genotype and plasma adiponectin levels were significant predictors of a high 10-year CVD risk. These observations indicate that apoE polymorphism may be a risk factor for dyslipidemias or CVD risk in RA patients. However, larger sample size will be needed to validate our findings in future studies. 

In this study, we revealed that the distribution of apoE genotypes between RA patients and HC was indistinguishable, and, similar to previous reports [34]; the majority of apoE the genotype was ε3/ε3. The correlation between plasma apoE levels and CVD risk is still controversial, and van Vliet et al. revealed a positive association of plasma apoE levels with stroke risk [35]. However, circulating apoE, a glycoprotein involved in lipid transport and metabolism [20], may play a protective role in the development of atherosclerosis and CVD in humans [36]. The anti-atherosclerotic and anti-inflammatory capacity of circulating apoE may explain our results of significantly higher apoE levels in our patients with the ε2ε3 genotype who had low CVD risk when compared with ε3ε3 or ε3ε4 genotype carriers.

Dyslipidemia is a well-established traditional risk factor for atherosclerosis and CVD [37]. Similarly to the results of the previous reports [33,38,39] showing that subjects carrying the ε2 allele have lower cholesterol levels compared with carriers of other alleles, we revealed significantly lower LDL-C levels and AI scores in RA patients with ε2ε3 genotype compared with ε3ε4 patients. Given a positive association of LDL-C levels and AI scores with CVD risk, our results support the accumulating evidence that the ε2ε3 genotype is associated with low CVD risk, whereas the ε3ε4 genotype is a risk factor of CVD [40,41].

There are several shared characteristics between atherosclerosis and RA. Elevated CRP level is associated with disease severity and future cardiovascular events in both conditions. Accordingly, our patients with ε2ε3 genotype had significantly lower CRP levels compared with carriers of ε3ε4 genotype who had high CVD risk. Our results were also consistent with previous reports that apoE genetic variants were associated with CRP levels in the general population [42] and RA patients [38]. Given a positive association of CVD risk with TNF-α and IL-6 [12,13], our patients with ε2ε3 genotype had significantly lower levels of both cytokines compared with carriers of ε3ε3 genotype. Resonating with these findings, our RA patients with ε2ε3 genotype had significantly lower Qrisk-2 scores for the 10-year CVD risk compared with ε3ε3 genotype or ε3ε4 genotype. Taking these findings together, we could speculate that apoE genotypes may predict the risk for dyslipidemia and CVD in the RA cohort [33,37,38].

Similar to the findings of previous reports [17,43], our RA patients had significantly higher levels of plasma adipokines, including adiponectin, leptin, resistin, and visfatin, compared with healthy subjects. Adipokines are also associated with dyslipidemia or CVD risk [43,44]. With an insulin-sensitizing effect [45], adiponectin is positively associated with plasma HDL-C levels and protects against atherosclerosis [46]. The adiponectin levels were positively correlated with HDL-C levels and negatively associated with insulin resistance in our RA patients with ε3ε3 genotype. In logistic regression, plasma adiponectin levels were a negative predictor of a high 10-year CVD risk in our RA cohort. Similar to the findings reported by Yadav et al. [47], plasma leptin levels were positively correlated with IR and TG levels in our patients with ε2ε3 genotype. Besides its putative role in IR, resistin has recently been found to promote the formation of foam cells and atherosclerosis [48]. There was a positive correlation between plasma resistin levels and IL-6 levels, contributing to increased IR in RA [49]. Our patients with the ε2ε3 genotype also had significantly lower resistin levels compared with ε3ε3 genotype. Furthermore, visfatin’s facilitating effect on adipogenesis [50] supports a positive link between visfatin levels and IR in our patients with ε3ε3 genotype. Although visfatin could promote atherosclerotic process and carotid plaque destabilization, Robinson et al. reported an association of visfatin expression with reduced CVD risk in RA patients [51]. Hence, the pathogenic role of visfatin in CVD risk in RA needs to be further validated. Based on these observations, we may speculate that both apoE genotypes and adipokine levels are related to CVD risk in the RA population. 

The relationship between apoE genotypes and adipokines in RA patients had never been reported. A prior report in postmenopausal women with apoE polymorphism demonstrated that women with ε4ε4 genotype exhibited the lowest leptin levels compared with ε2ε3, ε3ε3, and ε3ε4 genotypes [52]. Moreover, adiponectin levels among apoE genotypes were comparable. Our results showed higher leptin but lower resistin and visfatin in RA patients with ε2ε3 genotype compared with ε3ε3 genotype. Due to a small case number, ε4ε4 genotype RA patients were excluded from the analysis. Further study with a larger cohort is needed to explore the relationship between apoE and adipokines in RA.

During an at-least-5-year longitudinal follow-up, 17 (11.3%) of our RA patients developed newly diagnosed CVD (11 ischemic strokes, six acute myocardial infarction). Patients with ε3ε4 genotype tended to have a higher proportion of CVD compared with the ε2ε3 genotype carriers (17.9% versus 5.3%). Other studies also found the presence of ε3ε4 genotype or ε4 allele to be associated with high risk, while the ε2 allele with low risk, of CVD [40,53,54]. Resonating with previous reports [55,56], we further showed the ε3ε4 genotype as a positive predictor of a high 10-year CVD risk by using multivariate regression analysis. However, our findings should be verified by more extensive studies due to the use of many variates in this small patient sample.

Despite the novel findings in this pilot study, there were still some limitations. First, the sample size of RA patients in whom we observed CVD emergence was small and may result in decreased statistical power. Because corticosteroids and disease-modifying anti-rheumatic drugs (DMARDs) may influence plasma levels of apoE, adipokines, or inflammatory cytokines [57], their interference should be considered. Moreover, lipid profiles and CVD risks may have changed following conventional synthetic (cs)DMARDs and biological therapy [58,59]. A longitudinal study design might be needed to address how DMARDs therapy was associated with changes of apoE, adipokines, inflammatory cytokines, and CVD risks in RA patients with different apoE genotypes. In addition, none of the enrolled patients in our study were in the early RA stage, limiting the generalizability of these results to the whole population. Long-term research that enrolls more RA patients, including the early RA population, is needed to confirm these data. 

## 5. Conclusions

RA patients carrying ε2ε3 genotype, who have lower levels of TNF-α, IL-6, resistin, and visfatin, may have lower CVD risk compared with ε3ε3 genotype carriers, while those with ε3ε4 genotype, who have higher levels of LDL-C and IR, may have higher CVD risk compared with ε2ε3 genotype carriers. These findings provide aid in making personalized therapeutic decisions to reduce CVD risk in RA patients.

## Figures and Tables

**Table 1:** Demographic data and laboratory findings in rheumatoid arthritis (RA) patients and healthy controls (HC) ^§^.

	RA (n = 150)	HC (n = 48)
Mean age (years)	52.8 ± 11.6	51.4 ± 7.3
Female (%)	123 (82.0%)	36 (75.0%)
Disease duration (years)	15.5 ± 10.2	-
DAS-28 at study entry	4.85 ± 1.27	-
Smoking (ever) (%)	30 (20.0%)	6 (12.5%)
Apolipoprotein E, ng/mL	2885.5 (1967.2–3731.2) *	2341.3 (2027.4–3117.9)
Adiponectin, levels, ng/mL	4629.8 (2593.9–6714.7) **	3191.1 (1817.0–4143.2)
Leptin levels, ng/mL	44.4 (18.6–71.8) *	37.3 (13.1–47.1)
Resistin levels, ng/mL	7.3 (4.7–15.7) **	4.7 (2.9–9.9)
Visfatin levels, ng/mL	113.4 (94.3–131.2) *	94.8 (79.9–116.0)
TNF-α levels, pg/mL	31.9 (12.7–56.4)	28.1 (10.0–36.5)
Interleukin-6 levels, pg/mL	101.2 (56.3–389.0)	96.9 (53.5–205.5)
Interleukin-17A levels, pg/mL	110.2 (63.9–176.4) **	68.2 (36.1–114.8)
Daily steroid dose, mg	6.4 ± 2.6	-
csDMARDs alone	99 (66.0%)	
Methotrexate dose, mg per week	9.0 ± 5.7	-
TNF inhibitors	33 (22.0%)	-
Rituximab	7 (4.7%)	-
Tocilizumab	11 (7.3%)	-

^§^ Data are presented as mean ± standard deviation, number (percentage) or median (25th–75th quartile). TNF-α: tumor necrosis factor-α. DAS28: 28-joint disease activity score. csDMARDs: conventional synthetic disease-modifying antirheumatic drugs. * *p* < 0.05, ** *p* < 0.005, versus HC, the Mann–Whitney U test was used for between-group comparison of numerical variables.

**Table 2:** Demographic data and laboratory findings at study entry in RA patients with different apoE genotypes.

	ε2ε3 (n = 19)	ε3ε3 (n = 103)	ε3ε4 (n = 28)
Mean age (years)	56.7 ± 12.1	55.5 ± 14.1	58.1 ± 14.3
Female (%)	17 (89.5%)	83 (80.6%)	23 (82.1%)
Disease duration (years)	15.3 ± 5.0	14.4 ± 6.1	13.4 ± 7.8
RF positivity (%)	11 (57.9%)	77 (74.8%)	19 (67.9%)
ACPA positivity (%)	13 (68.4%)	70 (68.0%)	18 (64.3%)
Smoking (ever) (%)	2 (10.5%)	22 (21.4%)	6 (21.4%)
HT (%)	8 (42.1%)	53 (51.5%)	14 (50.0%)
DM (%)	2 (10.5%)	17 (16.5%)	6 (21.4%)
Body mass index, kg/m^2^	24.0 ± 3.03	24.1 ± 3.01	23.5 ± 2.33
Insulin resistance (HOMA)	1.83 ± 1.25	2.55 ± 2.90	4.05 ± 7.32
CVD event (%)	1 (5.3%)	11 (10.7%)	5 (17.9%)
Qrisk-2 score	4.25 ± 3.45 *	7.28 ± 8.49	8.76 ± 11.2
Total cholesterol, mg/dL	185 (159–214)	198 (173–227)	185 (172–217)
HDL-C, mg/dL	72 (60–80)	67 (57–88)	59 (53–74)
LDL-C, mg/dL	95 (89–124) ^#^	120 (96–146)	122 (103–141)
Atherogenic index	2.44 (2.27–2.76) ^#^	2.84 (2.36–3.43)	3.06 (2.77–3.63)
Triglyceride, mg/dL	83 (66–133)	96 (67–122)	83 (67–126)
TNF-α levels, pg/mL	9.4 (6.8–45.9) *	36.2 (24.4–66.0)	30.4 (8.9–48.8)
IL-6 levels, pg/mL	60.9 (53.6–101.2) *	115.1 (60.3–505.4)	93.1 (44.4–224.0)
IL-17A levels, pg/mL	109.3 (56.3–124.6)	116.6 (65.0–241.8)	87.4 (58.1–132.2)
CRP, at entry, mg/dl	0.42 ± 0.54 ^#^	1.24 ± 2.72	1.40 ± 1.45 *
DAS-28 at study entry	3.95 ± 1.34	4.19 ± 1.39	4.65 ± 1.36
Apolipoprotein E, ng/mL	3622 (3163–5433) *^##^	3000 (2167–3931)	1913 (1466–2735) ***
Adiponectin, levels, ng/mL	4870 (2520–8104)	4417 (2465–6650)	4630 (3391–6640)
Leptin levels, ng/mL	58.3 (34.9–85.5) **	28.3 (13.2–70.5)	49.2 (26.8–72.0)
Resistin levels, ng/mL	4.2 (3.0–8.2) **	9.0 (5.7–17.4)	6.9 (4.4–10.9)
Visfatin levels, ng/mL	81.1 (61.6–120.7) **	119.0 (100.4–137.4)	114.3 (92.1–125.7)
Daily steroid dose, mg	3.4 ± 2.5 ^#^	4.2 ± 2.7	5.1 ± 2.8
The used DMARDs at entry			
csDMARDs alone	14 (73.7%)	67 (65.0%)	18 (64.3%)
TNF inhibitors	2 (10.5%)	25 (24.3%)	6 (21.4%)
Rituximab	1 (5.3%)	5 (4.9%)	1 (3.6%)
Tocilizumab	2 (10.5%)	6 (5.8%)	3 (10.7%)

RA: rheumatoid arthritis; ApoE: apolipoprotein E; RF: rheumatoid factor; ACPA: anti-citrullinated peptide antibodies; HT: hypertension; DM: diabetes mellitus; HOMA: Homeostasis Model Assessment; CVD: cardiovascular disease; HDL-C: high-density lipoprotein cholesterol; LDL-C: low-density lipoprotein cholesterol; Atherogenic index corresponds to the ratio of total cholesterol/HDL-C; TNF-α: tumor necrosis factor-α; IL-6: interleukin-6; CRP: C-reactive protein; DAS28: disease activity score for 28-joints; csDMARDs: conventional synthetic disease-modifying anti-rheumatic drugs; TNF-α: tumor necrosis factor-α. * *p* < 0.05, ** *p* < 0.01, *** *p* < 0.001, versus patients with apoE ε3ε3 genotype, determined by Mann–Whitney U test. ^#^ *p* < 0.05, ^##^ *p* < 0.001, versus patients with apoE ε3ε4 genotype, determined by Mann–Whitney U test.

**Table 3:** Correlations between adipokines and inflammation or lipid profiles in RA patients with different apoE genotypes.

ApoE ε2ε3 Genotype (n = 19)	Adiponectin	Leptin	Resistin	Visfatin
Disease duration	0.469	0.029	0.374	−0.060
CRP levels	0.433	0.345	0.391	0.352
TNF-α levels	0.345	0.552 *	0.427	0.421
IL-6 levels	−0.130	0.252	0.527 *	−0.118
IL-17A levels	−0.140	0.527 *	0.176	−0.056
Total cholesterol levels	0.053	0.280	−0.215	0.321
Triglyceride levels	0.044	0.678 **	−0.255	0.159
HDL-C levels	0.181	−0.139	0.016	−0.280
LDL-C levels	0.202	0.375	−0.195	0.504
Atherogenic index	−0.182	0.469	−0.047	0.509 *
Insulin resistance (HOMA)	−0.181	0.657 *	−0.044	0.220
Body mass index	−0.059	0.074	−0.315	0.048
**ApoE ε3ε3 Genotype (n = 103)**	**Adiponectin**	**Leptin**	**Resistin**	**Visfatin**
Disease duration	0.145	0.091	0.177	0.086
CRP levels	−0.242	−0.140	−0.153	0.173
TNF-α levels	−0.061	0.062	0.075	0.238
IL-6 levels	0.100	0.216	−0.115	0.237
IL-17A levels	0.326 *	0.245	−0.153	0.105
Total cholesterol levels	0.280	0.149	0.137	0.014
Triglyceride levels	−0.139	−0.042	−0.255	0.272
HDL-C levels	0.297 *	0.251	0.177	−0.174
LDL-C levels	0.171	−0.028	0.136	0.098
Atherogenic index	−0.093	−0.204	0.020	0.149
Insulin resistance (HOMA)	−0.395 *	−0.114	−0.097	0.349 *
Body mass index	−0.036	−0.024	−0.044	0.302 *
**ApoE ε3ε4 Genotype (n = 28)**	**Adiponectin**	**Leptin**	**Resistin**	**Visfatin**
Disease duration	0.287	−0.175	0.227	0.317
CRP levels	−0.014	0.284	0.090	−0.032
TNF-α levels	−0.044	0.886 **	−0.334	0.096
IL-6 levels	0.186	0.325	−0.138	−0.343
IL-17A levels	0.213	0.064	0.076	0.234
Total cholesterol levels	0.244	−0.193	0.052	0.290
Triglyceride levels	−0.003	−0.062	0.192	0.110
HDL-C levels	0.366	−0.111	0.141	0.433
LDL-C levels	0.145	−0.002	−0.065	0.109
Atherogenic index	−0.132	0.006	−0.074	−0.318
Insulin resistance (HOMA)	−0.311	0.020	0.560 *	0.325
Body mass index	−0.270	0.227	0.355	0.041

RA: rheumatoid arthritis; apoE: apolipoprotein E; CRP: C-reactive protein; TNF: tumor necrosis factor; IL: interleukin; HDL-C: high-density lipoprotein-cholesterol; LDL-C: low-density lipoprotein-cholesterol; Atherogenic index corresponds to the ratio of total cholesterol/HDL-C. * *p* < 0.05, ** *p* < 0.01, determined by using the nonparametric Spearman’s correlation test.

**Table 4:** Logistic regression analysis to predict a high CVD risk, defined by Qrisk scores ≧ 10.

Risk Factors (Univariate)	β-Value	95% Confidence Interval	*p*-Value
Constant			
ApoE genotype			
ε2ε3	Reference	Reference	
ε3ε3	3.03	(−1.22, 7.28)	0.162
ε3ε4	4.51	(−0.55, 9.58)	0.080
ACPA			
Negativity	Reference	Reference	
Positivity	3.58	(0.65, 6.51)	0.017
TNF-α levels, pg/mL	−0.0010	(−0.0033, 0.0024)	0.755
IL-6 levels, pg/mL	0.0003	(−0.0006, 0.0012)	0.495
IL-17A levels, pg/mL	−0.0005	(−0.0055, 0.0044)	0.828
Adiponectin, levels, ng/mL	0.0006	(0.0002, 0.0010)	0.007
Leptin levels, ng/mL	0.005	(−0.021, 0.030)	0.722
Resistin levels, ng/mL	0.04	(−0.05, 0.12)	0.403
Visfatin levels, ng/mL	0.02	(−0.02, 0.07)	0.229
Atherogenic index	3.06	(1.52, 4.60)	<0.001
**Risk Factors (Multivariate)**	**β-Value**	**95% Confidence Interval**	***p*-Value**
ApoE genotype			
ε3ε4	4.86	(0.62, 9.10)	0.025
ACPA			
Positivity	3.17	(0.08, 6.25)	0.045
Adiponectin, levels, ng/mL	0.0005	(0.0001, 0.0010)	0.012

ApoE: apolipoprotein E; ACPA: anti-citrullinated peptide antibodies; TNF: tumor necrosis factor; IL: interleukin; Atherogenic index corresponds to the ratio of total cholesterol/HDL-C.
